# Kinetic Energy Harvesting for Wearable Medical Sensors

**DOI:** 10.3390/s19224922

**Published:** 2019-11-12

**Authors:** Petar Gljušćić, Saša Zelenika, David Blažević, Ervin Kamenar

**Affiliations:** 1 Faculty of Engineering, University of Rijeka, Vukovarska 58, 51000 Rijeka, Croatia; pgljuscic@riteh.hr (P.G.); ekamenar@riteh.hr (E.K.); 2 Centre for Micro- and Nanosciences and Technologies, University of Rijeka, Radmile Matejčić 2, 51000 Rijeka, Croatia; 3 Faculty of Information Technology and Communication Sciences, Tampere University, Korkeakoulunkatu 3, 33720 Tampere, Finland; david.blazevic@tuni.fi

**Keywords:** kinetic energy harvesting, wearable medical sensors, coupled electromechanical analysis, optimized design configurations, frequency bandwidth, energy management

## Abstract

The process of collecting low-level kinetic energy, which is present in all moving systems, by using energy harvesting principles, is of particular interest in wearable technology, especially in ultra-low power devices for medical applications. In fact, the replacement of batteries with innovative piezoelectric energy harvesting devices can result in mass and size reduction, favoring the miniaturization of wearable devices, as well as drastically increasing their autonomy. The aim of this work is to assess the power requirements of wearable sensors for medical applications, and address the intrinsic problem of piezoelectric kinetic energy harvesting devices that can be used to power them; namely, the narrow area of optimal operation around the eigenfrequencies of a specific device. This is achieved by using complex numerical models comprising modal, harmonic and transient analyses. In order to overcome the random nature of excitations generated by human motion, novel excitation modalities are investigated with the goal of increasing the specific power outputs. A solution embracing an optimized harvester geometry and relying on an excitation mechanism suitable for wearable medical sensors is hence proposed. The electrical circuitry required for efficient energy management is considered as well.

## 1. Introduction

Energy harvesting is the process of collecting low-level ambient energy and converting it into electrical energy to be used as a power source for miniaturized autonomous devices. Examples of this can be seen in structural health monitoring, smart packaging solutions, communication systems, transportation, air and aerospace vehicles, structural biology, robotics, microelectromechanical systems (MEMS) devices, sensor networks, wearable electronics, agriculture, forest fire detection, or various Internet of Things (IoT) components [1,2,3,4,5,6,7,8,9]. Examples of successfully demonstrated possible applications are tire pressure monitoring systems, resulting in autonomous devices powered by the motion of the vehicle [10], or the measurement of river pollution via autonomous sensor nodes powered by the river flow itself [11].

A growing field of the application of energy harvesting technologies are ultra-low power autonomous wearable sensors, e.g., heartbeat, body temperature, blood pressure, blood sugar level or acceleration (e.g., in the case of fall detection) sensors, used in remote health monitoring and telemedicine. Such devices could greatly benefit from the replacement of batteries with an energy harvesting device, thus allowing the reduction of their dimensions and masses, while more importantly, achieving increased autonomy levels [3].

An important issue related to the medical applications of IoT systems is privacy and data protection [12]. The main concerns in this framework include the integrity of acquired data, its usability and auditing, as well as the privacy of patient information. Potential solutions suggested in literature generally comprise, in turn, several possible data encryption and control approaches, trusted third party auditing of the acquired data, as well as data anonymization via the usage of identifiers such as ID numbers, names or phone numbers [12].

To explore the possible application of energy harvesting technologies for wearable medical sensors, in Section 2 of this work the power requirements of such sensors, with the associated data logging and transmission elements, are considered. This constitutes the basis for developing appropriate energy harvesting devices apt to provide the needed power.

The ambient energy sources generally considered for energy harvesting comprise solar (light) energy, radio-frequency, kinetic energy and waste heat [2,3,13]. Kinetic energy, pervasive in the environment, is generally caused by the motion of living beings or machinery, which makes it an especially interesting energy harvesting source for autonomous, remote and wearable applications. Of the several possible methods utilized to date to convert kinetic into electrical energy, piezoelectric transducers have proven to be advantageous due to design simplicity, miniaturization and integration potential, as well as high energy density [14]. The primary objective in designing piezoelectric energy harvesting devices, considered in Section 3 and Section 4 of this work as a viable power source for medical wearable systems, is to achieve maximum efficiency for a given application within the existing spatial limitations [1,2,14]. An inherent drawback in commonly-used piezoelectric harvesters is, however, that the highest achievable voltage and power outputs are within a narrow area around the eigenfrequency of a specific device, while the output values rapidly decrease with even a minor variation of the excitation frequency [1,2]. A thorough review of the potential solutions for this problem is given in Section 4, where are given also some guidelines on the power management electronics to be preferably coupled to the resulting optimized design configurations of the harvesters.

So far, a systematic study aimed at identifying the power requirements of wearable sensors and respective data elaboration and transmission systems, and especially at optimizing the design configuration of a piezoelectric kinetic energy harvesting device for powering such sensors, has not been produced. The aims of this work are, therefore, particularly:

- To address this problem by using coupled numerical analyses, experimental characterizations and novel excitation modalities;
- To propose a modular design of a harvester that enables increasing the attainable specific power outputs while overcoming the limitations induced by the random nature of excitations generated by human motion, and;
- To suggest a generalized scheme of electrical circuitry necessary for the corresponding energy management.

## 2. Power Requirements of Wearable Medical Sensors

The term “wearable technologies” commonly encompasses miniaturized electronic devices that can be worn on the human body as a part of clothing or as a distinct accessory, e.g., a watch or a wristband, or in the form of implants. Wearable devices may include a considerable variety of sensors, as well as data processing and communication elements, enabling a large diversity of possible applications. Several areas can considerably benefit from the implementation of such technologies, where some of the most prominent ones are very often related to health condition monitoring [15]:

- **Medicine**: patient health monitoring and early detection of disorders allowing timely medical interventions;
- **Risky Professions**: monitoring of the workers´ state to prevent dangerous situations or potential injuries, particularly common in construction, mining or shipbuilding;
- **Education**: stress level and health condition monitoring can provide a suitable foundation for the development of personalized learning plans, time management recommendations, or for scheduling of classroom activities;
- **Office Environment and Industry**: Occupational stress can cause the deterioration of health conditions, implying that the monitoring of the health parameters of the employees can be beneficial in preventing such occurrences;
- **Sports and Recreation**: Monitoring of parameters related to training activities and health conditions allows the prevention of injuries, achieving optimal fitness levels or assessing sleep quality.

Wearable technologies are commonly based on one or several sensors, a signal processor unit (in some instances accompanied with memory elements able to store data), power supply elements and wireless communication modules. In health monitoring applications, as well as in telemedicine, typical sensors may include accelerometers, sound and temperature sensors, heart rate monitors, pulse oximeters, as well as blood pressure and glucose level monitors [16,17,18,19,20,21,22,23,24,25,26,27,28,29]. Table 1 lists several variants of the mentioned wearable components, with the typical ranges of their power requirements, which constitutes an essential guideline for the development of the needed energy-harvesting devices that, when coupled to appropriate power management electronics, would enable their efficient use.

When considering the application of wearable systems in health condition monitoring or telemedicine, certain standards and guidelines should be taken into account. In fact, the measurement of different vital signs and health parameters is not performed in the same way or in the same intervals, which could have a significant impact on the power management of the whole wearable system. Although the majority of sensors consume a low amount of power (few tens of μW to, in the worst cases, a couple of mW), other components, such as signal processors and communication devices, could require higher power levels. This implies that the minimization of data transfer intervals, according to an appropriate medical practice, could lead to a better power management approach, as well as to improved system autonomy.

In this frame, the usage of accelerometers for fall detection in elderly or epileptic patients could be performed in a way that the data is sent only if the acceleration exceeds a certain threshold, i.e., in the case of a sudden change caused by a fall. According to medical guidelines, body temperature is commonly measured in patients in the morning and in the evening, which eliminates the need for constant data transfer, i.e., so called high power bursts are needed a couple of times a day for a very short period of time [11]. Heart rate is, in turn, usually measured continuously, so as to detect arrhythmia or other irregularities. A wearable device could, in this case, send an alarm signal only in case of a positive detection of values larger (or smaller) than a predefined threshold, thus eliminating the need for constant communication, and consequently reducing power consumption. Blood pressure is most commonly monitored a couple of times per day, so as to perform therapy corrections. Blood pressure data could thus be generally measured and transferred only in precisely defined intervals, allowing the sensor and communication components to operate on standby or be turned off in the meantime, further reducing power consumption. The monitoring of blood glucose levels is performed constantly in order to establish its levels during the day, especially before and after meals, to avoid the danger of hypoglycemia. The used sensor, integrated in a wearable system, could therefore perform a constant measurement of blood glucose levels and analyze the measured data, but the patient or the doctor should be alarmed only if the state of hypoglycemia occurs, limiting the usage of communication components. Finally, a pulse oximeter constantly measures the saturation of blood with oxygen, with the purpose of alarming the patient or doctor in the case of any low saturation, which can endanger the patient´s life. An oximeter could thus have a similar duty cycle as the blood glucose level monitor, performing a constant measurement, but sending data only in case of values smaller than a predefined threshold oxygen saturation level. Obviously, the correct intervals and threshold values should be carefully tailored to the needs of every single patient in accordance with medical expertise [30].

The ongoing and herein described research, involving a correct application of the aforementioned sensors and monitoring methods, is performed in collaboration with the Clinical Hospital Center in Rijeka, Croatia, and hence primarily aimed not at a constant monitoring of the health states, but rather at providing an alarm system notifying the patient or the doctor if potentially harmful conditions occur.

The above analysis constitutes then the basis for developing suitable energy harvesters based on the piezoelectric kinetic harvesting principles. In order to achieve this goal, suitable mathematical approaches to the modeling of the behavior of this class of energy harvesting devices have to be thoroughly evaluated in order to provide the means of subsequently optimizing their design to match the above stated requirements.

## 3. Materials and Methods in Modeling the Behavior of Piezoelectric Kinetic Energy Harvesters

**Figure 1:**
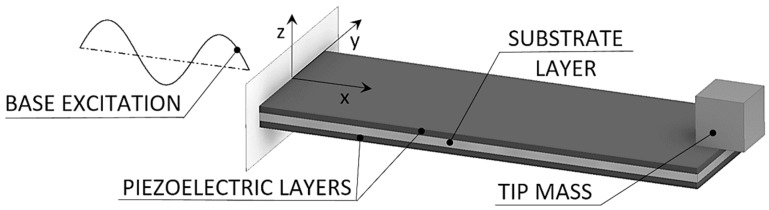
Piezoelectric bimorph cantilever.

A commonly used form of piezoelectric kinetic energy harvesting devices, thoroughly analyzed in this work, is the bimorph piezoelectric cantilever shown in Figure 1 [1,2,3]. Due to economic and technological reasons, the piezoelectric material used within this work corresponds to a commercially available PZT ceramic with the following main properties: density *ρ* = 7.8 g/cm^3^, Young’s modulus *E* = 65 GPa, piezoelectric coefficient $\bar{e_{31}}$ = −10.4 C/m^2^, permittivity constant $\varepsilon_{r\;33}^{S}$ = 830 and electromechanical coupling coefficient *k*_31_ = 0.3 [14,31]. On the other hand, the sizes of the used harvesters are different in the various used configurations, but to allow comparisons and relevant generalized conclusions, their respective performances are always normalized with respect to the geometrical parameters of the respective active piezoelectric layers.

The considered device comprises thus two layers of a piezoelectric material deposited onto a metallic substrate. The resulting cantilever is fixed on one end, while a tip mass, placed on its free end, amplifies the deflections and tunes the eigenfrequency of the device to the excitation frequency. In a dynamically-excited cantilever, mechanical energy resulting from the deformation of the piezoelectric layers is converted, via the piezoelectric electromechanical coupling effect, into electrical energy, generating a voltage difference between the electrodes deposited onto the surfaces of the piezoelectric layers [1,2]. In the following subsections, tools intended for modeling the dynamics of such devices are thoroughly described, evidencing their respective salient features, as well as the limits of their applicability.

### 3.1. Coupled Electromechanical Approach

In order to assess the response of different piezoelectric cantilever configurations of Figure 1, aimed at maximizing the obtainable voltage and power levels, suitable modeling algorithms are needed. Although several models able to assess the electromechanical behavior of the considered piezoelectric bimorphs are suggested in the literature [1,2,14], these are based upon lumped parameters, thus giving rise to potential inaccuracies [14]. A comprehensive “coupled modal electromechanical distributed parameter model” (CMEDM) was, in turn, recently developed [32], and it was shown that it is able to address the aforementioned inaccuracies inherent in previous simplified models [14]. 

It is based on solving the dynamics of the Euler-Bernoulli beam [33], while also taking into consideration the piezoelectric backward coupling effect (i.e., the fact that the electrical field in the piezoelectric material influences the mechanical response), the influence of the tip mass as well as the damping effects due to internal friction, and to the influence of the medium surrounding the harvester. The importance and the entity of the stiffening induced by backward coupling will be thoroughly discussed below in relation to the used numerical models and the thus-obtained results.

The resulting output voltage amplitude *α_s_* of the piezoelectric energy harvester for a harmonic excitation can in this case be expressed as [32]:

(1) $$\alpha_{s}\left(\omega \right)=\frac{\sum_{r=1}^{\infty }\frac{j\omega \kappa_{r}\sigma_{r}}{\omega_{r}^{2}-\omega^{2}+j2\zeta_{r}\omega_{r}\omega }}{\frac{1}{R_{L}}+j\omega \frac{C_{\tilde{p}}}{2}+\sum_{r=1}^{\infty }\frac{j\omega \kappa_{r}\chi_{r}^{s}}{\omega_{r}^{2}-\omega^{2}+j2\zeta_{r}\omega_{r}\omega }}e^{j\omega t}$$

where *j* is the imaginary unit ($=\sqrt{-1}$), *ω* is the excitation frequency close to harvester’s eigenfrequency, which itself is *ω_r_*, and next, *κ_r_* is the forward coupling term, *σ_r_* is the translational component of the excitation, *ζ_r_* is mechanical damping, *R*_L_ is the external electrical load acting on the system, $C_{\tilde{p}}$ is the capacitance of the piezoelectric material and $\chi_{r}^{s}$ is the modal coupling term [32]. The average power output of the harvester, dissipated across the resistor, will then be given by:

(2) $$P_{\mathrm{av}}=\frac{{\left|\alpha_{s}\right|}^{2}}{2R_{L}}$$

**Figure 2:**
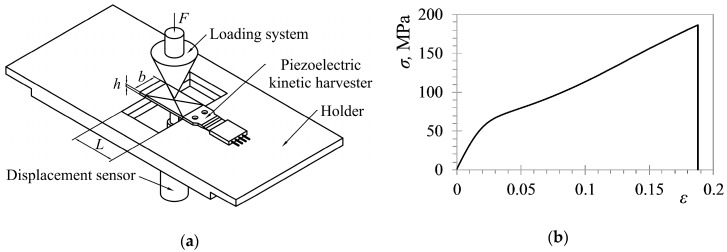
Two ways of determining the modulus of elasticity of multi-layered piezoelectric harvesters on a tensile machine: (**a**) after [34]; and (**b**) after [11].

In order to apply this model, as well as to tune properly the subsequently-used, finite element (FE) models, and for interpreting correctly the results of experimental measurements, it must be noted that off-the-shelf available kinetic energy harvesting cantilevers are generally multi-layered structures comprising two or more different layers. To obtain the eigenfrequencies of such structures, their equivalent bending stiffness (expressed in terms of the product of the respective Young’s Modulus with the second moment of inertia of the cross section of the harvester (*E*·*I*_z_)), needs to be determined. Tests have thus been set up on a tensile machine (Figure 2a) to measure the deflections of commercially-available harvesters while they are being subjected to a bending load. In the considered limited range of displacements, the measured load vs. the deflection data shows a linear behavior. Plate theory, i.e., the expression that correlates the modulus of elasticity of a simply supported plate to its dimensions, and to the deflections induced by centered bending point loads, hence allows the determination of the equivalent Young’s Modulus value [35]. Another approach is to use a conventional quasi-static tensile test to measure the Young’s Modulus of the multi-layered piezoelectric harvester from the resulting stress-strain curve (Figure 2b) [11]. 

**Figure 3:**
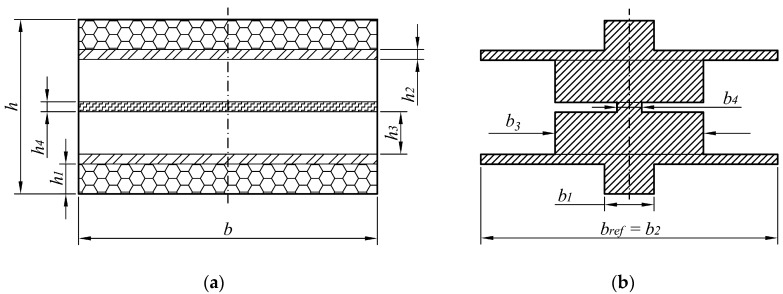
(**a**) Real and (**b**) Equivalent cross section of an off-the-shelf kinetic energy harvesting device with seven layers [36].

In either case, the experimentally-attained *E* values can be multiplied by the overall second moment of inertia of the cross section of the harvester, i.e., *I*_z_ = (*b*·*h*^3^)/12 (Figure 3a) in order to obtain the respective equivalent bending stiffness.

An alternative approach to the determination of the equivalent bending stiffness is to use the conventional strength of materials theory to convert the layered cross section of the harvester into an equivalent homogenous cross section (Figure 3b). The widths of the various sections are thus modified, in this case corresponding to the ratio of Young’s Modulus of that particular section to the modulus of the material chosen as the reference one; the respective distances of the considered sections from the neutral axis are, in turn, kept constant. The equivalent second moment of inertia of the cross section of the whole harvester can hence be obtained by considering the respective layer thicknesses, and taking into consideration Steiner’s Rule [35].

**Figure 4:**
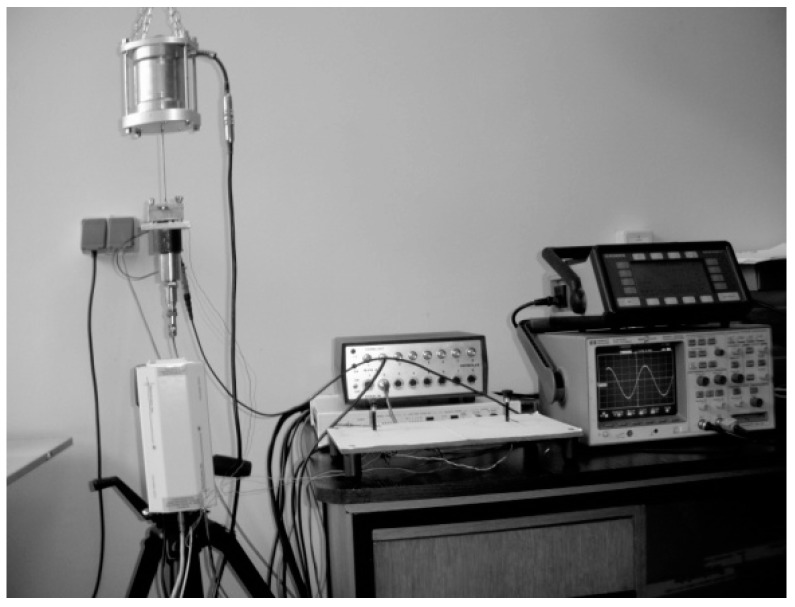
Experimental set-up for dynamical measurements [36].

Such approaches have been applied to an off-the-shelf class of piezoelectric kinetic harvesters (Figure 4), and the results attained via the CMEDM implemented in MATLAB^®^ [36,37] have been compared with those obtained experimentally on specifically developed set-ups at the Precision Engineering Laboratory of the Department of Mechanical Engineering Design of the Faculty of Engineering of the University of Rijeka, Croatia [38]. It has been shown that, in terms of the general trends related to the dynamical responses for variable electrical loads, as well as of the achieved peak voltages at a determined eigenfrequency, CMEDM provides reliable results for bimorph cantilevers with a constant rectangular cross-section, although there are residual discrepancies, probably due to nonlinearities (anticlastic effect [39], geometrically nonlinear deflections [40], compliance of the constraints) un-included in the CMEDM.

**Figure 5:**
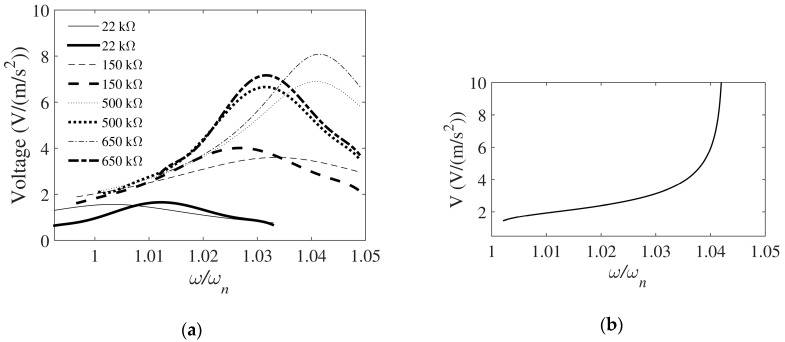
(**a**) Voltages obtained by employing the coupled modal electromechanical distributed parameter model (CMEDM) (thin lines) and experimentally (thick lines) for various *R*_L_ values; (**b**) Maximal voltages vs. *ω*/*ω_n_* for various *R*_L_ values attained via CMEDM [34].

From the frequency response functions (FRFs) of steady-state voltages vs. harmonic base acceleration (expressed as a ratio with respect to the uncoupled, i.e., pure mechanical, eigenfrequency *ω_n_*) for varying applied electrical loads *R*_L_ (Figure 5a), it can also be concluded that not only an increase of *R*_L_ causes a marked nonlinear increase of the amplitude of the maximal output voltages, but also that the influence of the backward piezoelectric effect on the dynamical response is significant. This hardening effect leads, therefore, to an increase >4% of the modal frequency where the output voltages are maximal, with respect to the uncoupled eigenfrequency of the same harvester (Figure 5b) [35].

**Figure 6:**
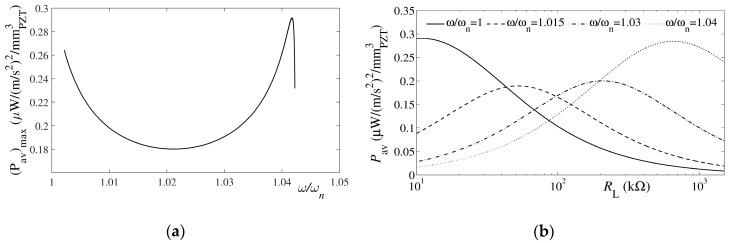
(**a**) Maximal average specific powers obtained by employing CMEDM for changing excitations and for varying *R*_L_; (**b**) Variation of CMEDM average specific powers vs. *R*_L_ (from short circuit to open circuit conditions) for different excitations [34].

Considering, in turn, the FRFs of the achieved average powers (normalized to the volume of the piezoelectric material) vs. base acceleration, a good correspondence of the general trends and the maximal values obtained experimentally and by using the CMEDM is obtained again, while the obtained dependence of the maximal powers vs. the normalized excitation levels is complex and not monotonic. In Figure 6a it is thus evident that, after an initial decrease of the maximal average power with increasing *R*_L_, an increase and then a secondary decrease occurs. This nonlinear dependency allows the optimal load (i.e., the load resistance allowing to attain the maximal power) to be determined for a specific piezoelectric kinetic harvester. In this frame, however, it has to be noted that several *R*_L_ values, depending on the excitation frequency, can result in the same value of the maximal average specific power. Considering then the whole theoretically possible range of loads applied to a specific harvester, the lowest *R*_L_ values will give maximal average specific powers for excitation frequencies corresponding to the short circuit condition, while the highest *R*_L_ values result in maximal specific powers for frequencies approaching the open circuit condition; intermediate excitation frequencies result, in turn, in smaller maximal specific average powers even for optimized *R*_L_ values (Figure 6b). What is more, for increasing *R*_L_ values a nonlinear hardening effect leads once more to an increase >4% of the modal frequencies where the values of the maximal specific powers are obtained [35].

All this confirms the postulated importance of backward coupling, implying that, for a specific excitation, only a matching of the design configuration of the harvester and of the applied load can allow the maximizing of the achievable power outputs. In any case, although the CMEDM allows, hence, an appreciation of the influence of the backward piezoelectric coupling on the dynamical response of the studied class of kinetic energy harvesting devices, as well as the intricate dependencies of the attained voltages and powers on the applied resistive loads, and determining the loads and frequencies where the powers can be maximized, all of this is achievable for piezoelectric cantilevers with a constant rectangular cross-section. When, in turn, shape variability is introduced as a viable design parameter, a cumbersome extension of the CMEDM model of a yet to be developed form would, however, become necessary.

### 3.2. Finite Element Approach

In order to investigate the influence of different cantilever geometries on the electromechanical response of piezoelectric kinetic harvesters, analyses of cantilevers with diverse and varying cross-sections are required. For that purpose, a more elaborate tool, based on comprehensive numerical coupled analyses by employing the finite element (FE) method, also allowing to take into consideration the stress concentration and charge distribution effects, is therefore developed and tuned with the experimentally-proven CMEDM. Such an approach enables a more efficient development of the innovative design configurations of the considered class of energy harvesting devices, resulting in optimal electromechanical responses for a given application. The employment of an FE approach hence provides a means of establishing how cantilever design parameters influence the response of the harvester and, in this manner, overcoming the limitations of CMEDM [14,37,41]. The complex electromechanical coupling occurring in the considered piezoelectric bimorphs requires, however, a complex 3D FE analysis approach comprising:

- Modal analysis allowing the determination of the mechanical dynamical response and the respective eigenfrequencies of the harvester;
- Coupled harmonic analysis resulting in coupled FRFs, and;
- Coupled linear and nonlinear transient analysis resulting in dynamical responses under forced excitation at discrete time steps, including geometrical nonlinearities.

The FE model is developed here using the ANSYS, Inc. (ANSYS^®^, Canonsburg, PA, USA) parametric design language (APDL) [14,31]. A basic multivolume 3D block model of the bimorph piezoelectric cantilever under harmonic base excitation is hence generated, and the respective material parameters are used. 

Standard ANSYS^®^ element types used for the modeling are:

- SOLID226 prismatic elements with 20 nodes and five degrees of freedom (DOFs) per node, enabling the simulation of piezoelectric material properties;
- SOLID186 prismatic elements with 20 nodes and three DOFs per node used to model the substrate and the tip mass;
- CIRCU94 element used in the harmonic and the transient analyses for the simulation of the electrical loads.

The implemented boundary conditions are equivalent to those used in the CMEDM model, i.e., the fixed end of the cantilever is clamped at the substrate layer, since clamping it at the piezoelectric layers would considerably shift the peak response towards higher frequencies [14]. It is worth noting here that this condition (clamping only the substrate) corresponds to the practical execution of the clamping in the experimental part of the work and in factual applications of piezoelectric kinetic energy harvesters, since, due to stress concentration effects in the considered dynamical (fatigue-related) applications, clamping of the unprotected PZT layers would lead to the damage and cracking of the PZT ceramics. For the same reasons (correspondence with the practical execution of the experiments), the tip mass is modeled so that its center coincides longitudinally with the free edge of the cantilever [31,37].

#### 3.2.1. Modal Analysis

The initial modal analysis is needed to prove the validity of the FE model via a comparison to the CMEDM, as well as a guideline for the subsequent frequency sweep in the harmonic analysis. In this work only the first modal shape is considered, since in practice, the first eigenfrequency allows the largest deformations and therefore the highest achievable output voltage values. A purely mechanical response of the bimorph, setting to zero the piezoelectricity coefficient in the material properties of the piezoelectric layers, is calculated, hence eliminating the effects of electromechanical coupling. According to ANSYS^®^ recommendations, instead of solvers that use a cumbersome iterative process, the sparse direct matrix solver, based on a direct elimination of equations, is used in these analyses, despite the resulting computational intensity, as it is the most robust solver type available in ANSYS^®^ [14,31,37].

**Figure 7:**
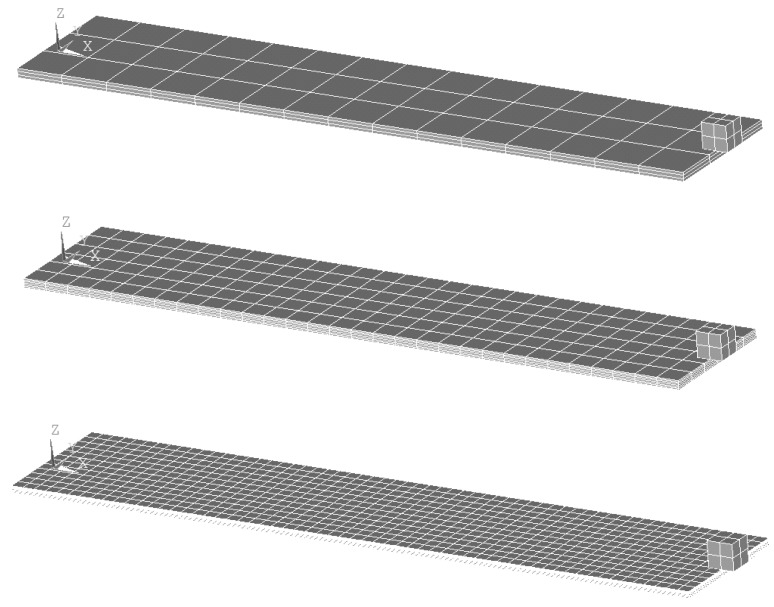
Increasing mesh densities (top to bottom) used in the performed analyses [31].

A mesh sensitivity analysis is performed next, using three mesh densities, each with a twofold increase in density with respect to the previous one (Figure 7), and the obtained results are compared with those obtained via the CMEDM model. It can thus be shown that, regardless of the considered mesh density, that the eigenfrequency resulting from the FE modal analyses results in negligible errors (<1%) with respect to the first bending eigenfrequency obtained via the CMEDM approach. This implies that, in general, FE modal analyses can be performed with a coarser mesh [34].

#### 3.2.2. Harmonic Analysis

To establish the coupled dynamical electromechanical response (coupled FRFs) of the considered design configuration of piezoelectric kinetic energy harvesters, coupled harmonic analyses are performed next. The considered frequency bandwidth of the harmonic excitation, in the form of a vertical sinusoidal acceleration of constant amplitude at the clamped base of the cantilever, is that around the eigenfrequency as determined from the performed modal analysis, while the other boundary conditions coincide with those used in the modal analysis. The displacements along the cantilever are thus obtained, allowing the charge distributions on the piezoelectric layers, and the resulting voltages and harvested powers, to be identified. Electromechanical coupling is achieved here by introducing a variable load resistance into the model, i.e., by inserting a CIRCU94 element between charge collecting nodes (resistor connectors) on the surfaces of the piezoelectric layers (thus simulating the respective electrodes) via electrical (VOLT) DOFs [31,37].

**Figure 8:**
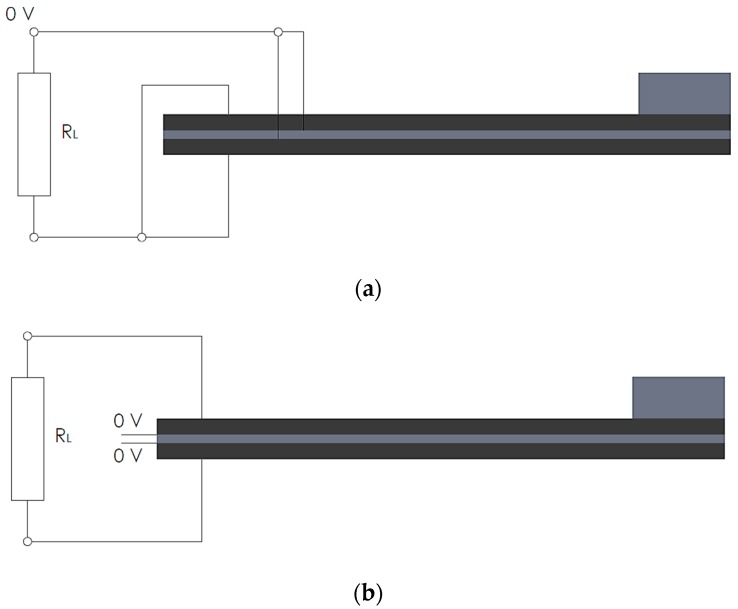
(**a**) Electrical connections on a parallel connection of the piezoelectric bimorph; and (**b**) respective serial connection.

As shown in [2], parallel or serial connection can be proficiently used in this frame, depending on the polarization of the piezoelectric layers. In the case of a parallel connection, the outer nodes of the piezoelectric layers are to be connected to one end of the resistor element, while the inner nodes (in contact with the metallic substrate) are connected to the other end, thus completing the circuit (Figure 8a). One of the load resistance nodes has to be connected to ground [31]. When, in turn, a serial connection is considered, the outer top nodes are connected to one end of the resistor, while the outer bottom nodes are connected to the other end. The nodes adjacent to the metallic substrate have to be connected to ground (Figure 8b) [42]. The output voltage is then measured on one of two nodes representing the connectors of the resistor.

It has to be noted here that the definition of damping, a complex phenomenon in distributed mechanical systems, is a major requirement in order to obtain accurate results of harmonic analyses. Rayleigh Damping, commonly used in FE analyses, comprises in this frame the calculation of the damping matrix ***B**_d_* as a sum of the mass ***M*** and stiffness ***K**_S_* matrices, multiplied by the corresponding damping constants *α* and *β* [31,37,43]:

(3) $$B_{d}=\alpha M+\beta K_{s}$$

By using the experimentally-determined damping coefficient *ζ*, along with the first two eigenfrequencies *f*_1_ and *f*_2_ from the previously-performed modal analysis, the damping constants *α* and *β* can hence be calculated from the following set of equations:

(4) $$\frac{\alpha }{4\pi f_{1}}+\beta \pi f_{1}=\zeta$$

(5) $$\frac{\alpha }{4\pi f_{2}}+\beta \pi f_{2}=\zeta$$

**Figure 9:**
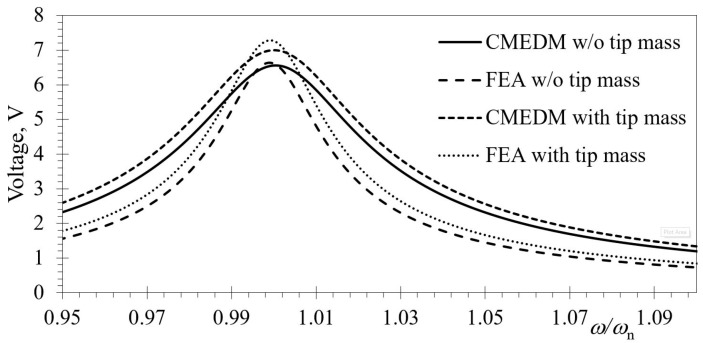
FE coupled electromechanical responses for a rectangular bimorph with and without tip mass compared to CMEDM results.

The optimal value of the *R*_L_ electrical resistive load (i.e., the one resulting in the highest power outputs) is then determined by performing a number of harmonic analyses while varying the *R*_L_ values in a range covering several orders of magnitude (from the Ω up to the MΩ range) [31,32,37]. From the comparison of the FRFs of harmonic analyses with those attained again by using CMEDM, an excellent correspondence is hence attained once more (with relative errors of maximal voltage outputs and respective eigenfrequencies <1%), confirming the suitability of the FE model in successfully forecasting the electromechanical coupling (including the backward coupling effect) and its influence on shifting modal frequencies (i.e., the hardening effect previously evidenced via CMEDM calculations) (Figure 9) [31,37].

**Figure 10:**
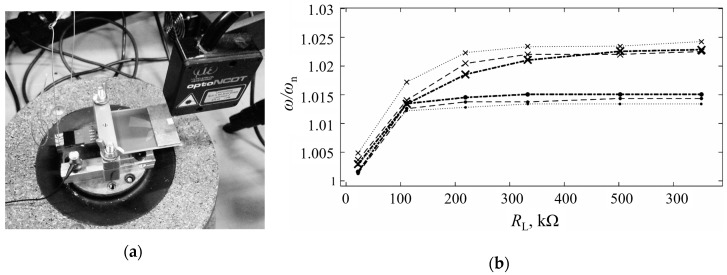
(**a**) Experimental set-up used to assess the performances of off-the-shelf piezoelectric kinetic harvesters; (**b**) Comparison of FE (dashed lines with “x” markers) and experimental (circular markers) results of the hardening effect for off-the-shelf piezoelectric kinetic harvesters with different tip masses [36].

The thus obtainable results for off-the-shelf piezoelectric kinetic harvesters are compared with corresponding experimental data, attained in this case at the collaborative Laboratory of Mechanics of the University of Udine, Italy (Figure 10a). 

By varying the excitation frequencies and the applied resistive loads, it is hence proven that the FE model allows a satisfactory (with relative errors generally <1%) prediction of the rise of the eigenfrequencies with increasing resistive loads induced by the backward coupling hardening effect (Figure 10b). The small visible deviations of the FE model results with respect to the experimental data can perhaps be attributed to previously evidenced ANSYS^®^ limitations in performing this type of simulation, due apparently to the theoretical formulation of the direct piezoelectric effect adopted in the ANSYS^®^ software package [41]. On the other hand, however, the voltage level discrepancies between the FE and the experimental data are larger, especially for larger tip masses and electrical loads, which requires a further thorough investigation [31].

#### 3.2.3. Linear and Nonlinear Transient Analyses

Transient analyses are finally performed in order to model the dynamical responses of the piezoelectric kinetic harvesters subjected to forced excitation in precisely defined discrete time increments, generating steady-state results for each time iteration [31,37]. In transient analyses, a sinusoidal excitation profile, generated in MATLAB^®^, is imported into ANSYS^®^ in tabular form and implemented in each time-step via a *DO loop, while a sufficiently large number of cycles is needed at each considered frequency to assure the fulfilment of steady-state conditions. Due to the time-consuming execution of each analysis step, the analyses are performed within a narrow range around the first eigenfrequency. The aforementioned damping coefficients *α* and *β* are, in turn, set again to the same values as in the harmonic analyses, whereas the first and second order transient integration parameters used in the ANSYS^®^ routines are set according to the ANSYS^®^ recommendations for piezoelectric analyses [31,37]. The 3D geometry of the bimorph cantilever, and the setting of the DOFs, of the coupling of the electrodes, as well as of the load resistance values, remain unchanged with respect to those used in the harmonic analyses.

It is especially important to note here that in various structures, e.g., shells, or as in this case, beams, the occurrence of large deflections (larger than ca. 5% of the cantilever’s length) causes the cross sections of the modeled structure to rotate with respect to each other. What is more, the stress-strain relationship might in this case take a nonlinear form, and the stiffness of the device might change, thus making the dynamical response dependent upon the excitation amplitude. In such cases the responses can thus no longer be predicted by the assumptions of the linearized Euler-Bernoulli Theory, but these nonlinear effects, that reasonably occur particularly when the piezoelectric kinetic harvesters are used in the vicinity of their resonant state, i.e., when the largest amount of mechanical energy is converted into electrical energy, have to be considered. 

The inclusion of these effects in the considered transient analyses is assured via the activation of the NLGEOM option, when for each time step ANSYS^®^ automatically takes into account the dependence of cantilevers’ stiffness on the reached positions of the nodes and recalculates the resulting stiffness matrix [31,33,37].

**Figure 11:**
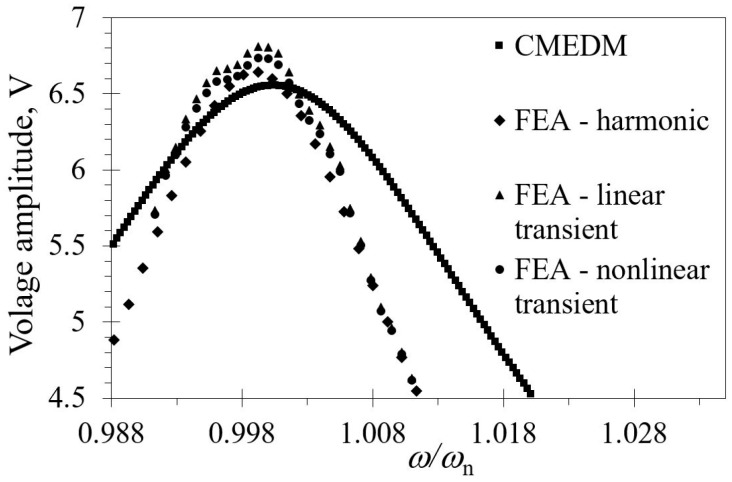
Linear and nonlinear FE transient responses for a rectangular piezoelectric bimorph compared with analytical CMEDM and FE harmonic responses.

In Figure 11 are shown the results of the performed FE linear and nonlinear transient analyses for a rectangular piezoelectric kinetic harvester. The depicted values are obtained by transforming the output of the calculations, obtained in the form of time-related voltage values, to the previously used FRF representations. It can thus be seen that the nonlinear analyses result in only slightly lower peak voltages, indicating that the contribution of the nonlinear effects in the overall dynamical behavior of the considered harvesters is rather limited. On the other hand, the obtained eigenfrequencies, as well as, within certain limits, the overall system responses in terms of the maximal achievable voltages, are essentially coinciding, not only among themselves, but also with those obtained by using the CMEDM approach and the harmonic FE analysis (cf. again Figure 11) [31,37]. The small “glitches” that are seen in the results of transient analyses in the vicinity of the peak voltages have, in turn, probably no physical foundation, since they have not been observed in any of the performed experimental measurements.

## 4. Piezoelectric Kinetic Energy Harvesters for Wearable Medical Monitoring Systems

As it is evident from the above shown results of the CMEDM and the FE numerical analyses, as well as from the depicted experimental results, the amplitude of the obtained voltages in the described design configuration of piezoelectric kinetic energy harvesters is highest within a narrow area around the eigenfrequency of a specific device, rapidly decreasing with even a minor variation of the excitation frequency. This phenomenon represents the major limitation of piezoelectric bimorph cantilevers used as energy harvesting devices in applications with variable excitations, such as is the kinetic energy of human motion, causing a drastic decrease of the energy conversion efficiency, as well as of the maximum possible voltage outputs [1,2]. Several approaches to solve this problem, i.e., to attain the broadening of the optimal frequency spectrum, have been suggested in recent literature [44]:

Changing the conditions around the cantilever free end (e.g., via damping control or active tuning); changing the geometry of the cantilever (by using complex geometries with bi-stable or nonlinear responses, or a large number of differently tuned cantilevers); and frequency up-conversion mechanisms, such as plucking the free end of the piezoelectric cantilever and letting it oscillate at its eigenfrequency.

A considerable amount of research focused on kinetic energy harvesting, with emphasis on the usage of kinetic energy caused by human motion to power wearable devices, has recently been carried out. Smilek et al. suggested a device comprising a rolling mass with permanent magnets able to collect low frequency kinetic energy, thus generating electrical energy, but lacking again the possibility of tuning the operating frequency [45]. Bai et al. analyzed the possibility of a piezoelectric device with four separate cantilevers and a common free mass to collect and convert kinetic energy caused by human motion. The research provided a much needed insight into the type and levels of available kinetic energy when the device is fixed to a specific area of the human body (e.g., hand, arm or head) in the laboratory as well as in real life conditions [46]. Xu et al. suggested a piezoelectric energy harvester with an electromagnetic active tuning system at the free end of the cantilever, allowing the broadening of the operating frequency bandwidth of the harvester, as well as the increase of the specific power output. The added tuning system increases, however, the complexity of the device, and requires an additional energy source to power the electromagnets [47]. Pozzi et al. studied a frequency up-conversion mechanism operating on the principle of plucking the free end of several cantilevers by plectra located on the rotating part of a mechanism affixed on the leg at the knee. The design of the energy harvesting device is such that it allows further improvements in terms of the structure of the piezoelectric elements as well as the frequency up-conversion mechanism itself [48]. The excitation of a piezoelectric energy harvester can also be achieved by using magnetic plucking, as shown by Xue et al. [49]. In this case, however, the possible damping of the free end, caused by the exciting magnets, could negatively affect the vibration amplitude and thus the achievable output power levels. The approach described by Moro and Benasciutti consists, in turn, of a piezoelectric energy harvester mounted inside the heel of a shoe, so as to collect the kinetic energy caused by walking. While the device is able to produce significant power levels, in order to efficiently use all the available space, an optimization of the structure would be needed [42]. It should also be noted that, in order to avoid mechanical damage of the piezoelectric layers, the mechanical properties of the piezoelectric ceramics should be considered if they are applied in areas under high impact stress (e.g., walking, running). Benasciutti et al. analyzed, finally, the influence of a variation of the cantilever’s geometry on the specific power output of a bimorph energy harvester. This study has shown that a trapezoidal and an inverse trapezoidal shape of the bimorph could induce a significant increase of the specific power outputs [14].

Based on the above-listed methods that can be used to broaden the frequency bandwidth of piezoelectric kinetic energy harvesting devices for medical sensors, an inventive combined approach to the innovative design of such devices is thus proposed. The basic suggested design principle is to change the geometry parameters of piezoelectric cantilevers, as well as to use concurrently the frequency up-conversion excitation mechanism. An appropriate combination of these design principles could lead to the design of a new class of piezoelectric kinetic energy harvesting devices complying with the power requirements of wearable medical devices evidenced in Section 2 of this work, while assuring the overcoming of the limitation of bimorph harvester configurations excited by random frequency movements, conforming with the ever-increasing miniaturization requirements for wearables, and coupling such devices with suitable power management electronics.

### 4.1. Frequency Up-Conversion

**Figure 12:**
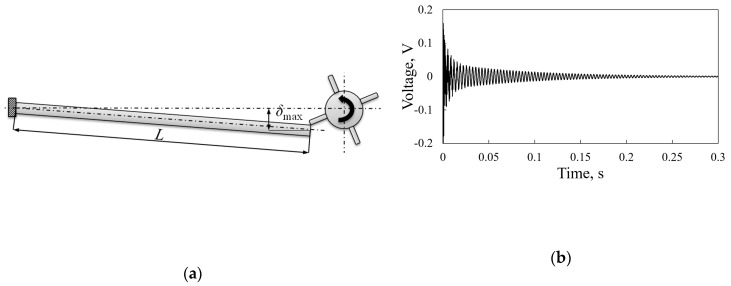
(**a**) Scheme of the frequency up-conversion principle induced by plucking; (**b**) Respective transient response [11].

The frequency up-conversion mechanism consists of plucking the free end of the piezoelectric kinetic harvester by plectra mounted on a rotating body and allowing it to oscillate freely at its eigenfrequency (Figure 12a). In this way, the harvester operates at its optimal working conditions, which results in the highest possible voltage (and power) outputs, allowing it to generate up to ca. 2 mW of power [48]. The transient response can in this case again be analyzed numerically by employing an ANSYS-based FE modeling of the deflection of the piezoelectric kinetic harvester resulting from the impact of the plectrum, while the respective excitation profile can be modeled via the MATLAB^®^ software package as an impulsive load inducing the free vibrating response shown in Figure 12b [11].

**Figure 13:**
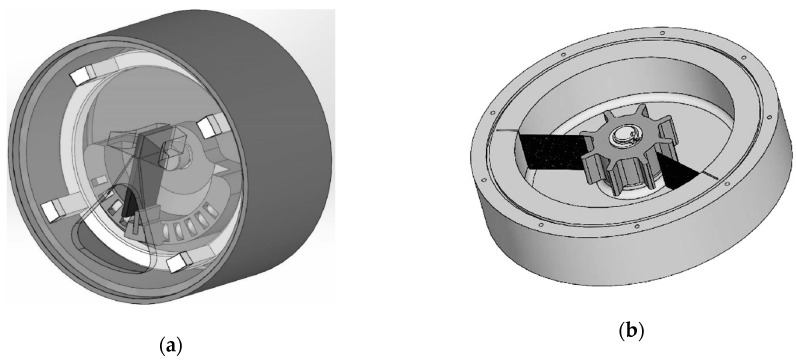
Proposed watch-like wearable devices based on frequency up-conversion [37].

Within the frame of the work on developing a harvesting solution for powering wearable medical sensors, two watch-like devices, operating on the frequency up-conversion principle, are hence studied in collaboration with medical institutions (cf. Figure 13a,b, respectively). The plucking motion is, in this case, generated by a rotating flywheel with several plectra mounted on a rotating hub, whose rotation is caused by the random movement of the hand and arm, which is thus transformed into a periodic excitation of the piezoelectric kinetic harvesters, as analyzed in Section 3 of this work [37,50].

### 4.2. Geometry Optimization

For cantilevers of equal maximal widths, it was recently proven, both numerically via FE analyses and experimentally, that the modification of the geometry of the piezoelectric kinetic harvester from a conventional rectangular to optimized trapezoidal shapes, allowing a near uniform stress distribution along the cantilever surface so that the piezoelectric material is elastically strained in every portion of the bimorph’s layer close to its strength limit, can lead to an increase of the resulting specific power outputs of up to 24% [14]. What is more, by inverting the trapezoidal shape, i.e., by clamping the trapezoidal piezoelectric kinetic harvester at its narrower end, due to the stress concentration effects in the vicinity of cantilever’s fixation, an increase in specific power output, compared to that of the rectangular form of equal maximal width, of up to event 113% can be achieved [14].

**Figure 14:**
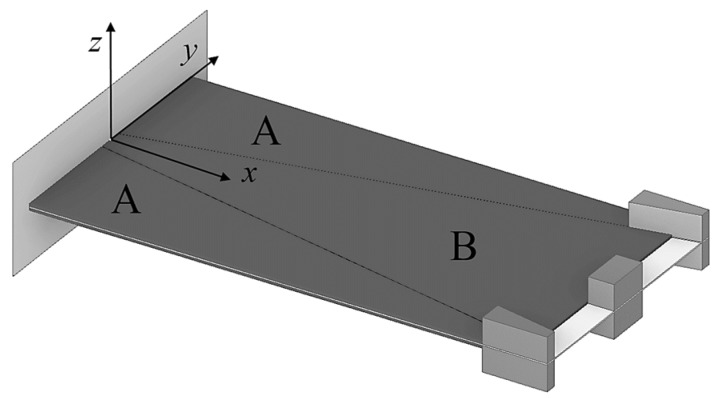
Segmented piezoelectric kinetic harvesters [50].

In order to maximize the power output of a piezoelectric kinetic wearable harvester with a predefined limited surface area, and thus reduce the overall size of the device considered in the frame of this work for powering medical sensors, the conventional rectangular surface of the harvester (indicated with “R”) is therefore divided in Figure 14 into two trapezoidal (A) and one inverse trapezoidal (B) segment [37,50]. The considered thicknesses of the substrate and of the piezoelectric layers, as well as the tip masses are in turn equal in all studied bimorphs.

**Figure 15:**
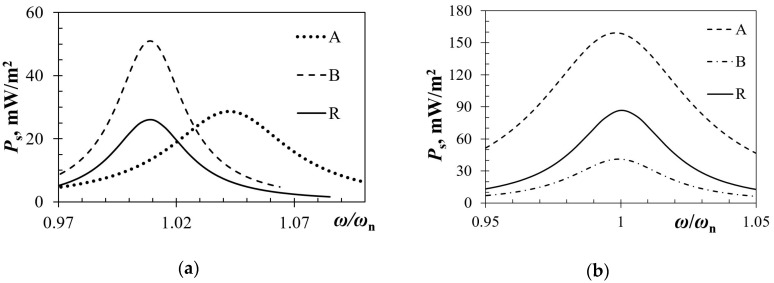
(**a**) FE results on the specific power outputs of the analyzed geometries; (**b**) Specific power outputs for segmented piezoelectric kinetic harvesters with optimized tip masses.

Since the piezoelectric kinetic harvesters are now intended to be excited by plucking, allowing each segment to oscillate at its eigenfrequency, a coupled harmonic FE analysis, thoroughly described in the above Section 3.2.2., is then performed for each segment separately. The optimal load resistance, allowing an achievement of the highest power output for each segment, is thus determined by sweeping through a spectrum of resistance values. In Figure 15a are hence depicted the maximal power outputs at the respective optimal loads for each segment. The specific power output values are obtained by normalizing the calculated powers with the surface area of the respective segment. From the attained data, it can be observed that segmenting the piezoelectric kinetic harvester results in higher specific power outputs with respect to the rectangular shape. Based on the data depicted in Figure 15a it should also be noted that, while each of the two trapezoidal segments designated as “A” has a somewhat higher specific power output than the original rectangular geometry, the specific power output of the inverse segment (“B”) is significantly higher. What is more, compared to the rectangular shape, the inverse segment “B” induces a slight eigenfrequency shift, while the shift for the trapezoidal segments “A” is more pronounced [50].

When, instead of using same tip masses on all the considered harvester’s geometries, the maximum allowable stress criterion for the piezoelectric material is used to optimize the tip mass for each segment; the obtained results shown in Figure 15b, compared to those in Figure 15a, show a clear increase of the specific power for the trapezoidal segments “A” (as well as “R”) and a decrease for the inverse trapezoidal segment “B”. This is mainly due to the narrow fixture of segment “B” that, inducing a stress concentration effect, which is beneficial in terms of the herein considered increase in charge generation, also causes a clear limitation in the possible tip mass value. It is in any case evident that the effect of tip mass on the power output is significant, and therefore, when aiming at maximizing the power outputs of the piezoelectric kinetic harvesters, there is a need to carefully perform a coupled optimization, considering concurrently the geometry of the harvester and the respective tip mass.

It is well worth noting here also that the geometrically-optimized configuration allows an interesting additional designing degree of freedom as well. In fact, by matching the maximal power output of each segment to a load equivalent to that of a specific wearable sensor, a further increase of the efficiency of the proposed solution with respect to those used up to date could be achieved. The variation of the tip mass on each segment can, in turn, enable the tuning of the respective eigenfrequencies to match the requirements of specific applications, thus providing a significant supplementary optimization potential.

In the specific case when the overall surface area of the set of segmented piezoelectric kinetic harvesters in Figure 14 is 20 × 40 mm, the respective absolute power levels, considering the optimal dynamical usage of all segments, would be around 500 µW. Assuming that the harvesters are used to power a temperature sensor, an accelerometer and a glucose monitoring sensor, and supposing a measurement duty cycle of up to 5%, with a data transmission duty cycle of 2 to 3 times per day (see the respective elucidations in Section 2), the harvested power should thus be sufficient to successfully achieve the foreseen operation of a factual medical system which has, however, to be proven by sound experimental data.

### 4.3. Power Management in Wearable Medical Monitoring Systems

The characteristic wearable devices listed in Table 1 require stabilized direct current (DC) power signals in order to operate properly. The typical operating voltages of such loads are converging to standardized values of 3.3 V or 5 V DC [16,17,18,19,20,21,22,23,24,25,26,27,28,29]. The voltage outputs from the energy harvesting devices, on the other hand, depend on the principle used to collect the low-level energy. In cases when, for example, the energy harvesting device uses a DC actuator as an active element [11], DC voltage with variable amplitude (depending on the velocity of actuator’s rotor) is present at its output. In the case herein considered, due to the excited bending of the cantilever bimorph induced by the kinetic energy of vibrations, the optimized segmented piezoelectric kinetic energy harvesters described in the above treatise generate at their outputs an alternating current (AC). This current could be characterized, depending also on the foreseen sensor powered by them, not only by varying amplitudes, but also by differing frequencies. Taking this into account, the harvested energy has to be properly managed to attain a smooth and stabilized voltage supply that can be interfaced to the considered load (medical sensor with signal processing and communication components). A further task designated to such electrical circuitry is to manage the surplus energy when it is produced, but the sensors and the respective data elaboration and transmission electronic components are in a dormant state, i.e., to store such energy on a suitable storage element (i.e., a capacitor [10], a rechargeable battery or a super-capacitor [11]), so that it can be efficiently delivered to the load when needed. By using the storage element, short power bursts can be achieved as well, i.e., high amounts of energy can be delivered to the load in short periods of time, as is commonly needed, especially for the data logging and data transmission components in some of the aforementioned wearable medical applications.

In general, the “core” element of a power management electronics set is a highly efficient DC-to-DC buck converter. The basic principle of the operation of any buck converter is to collect the low-level energy onto a low-capacity storage device (usually a capacitor) on the primary side, and “transfer” this energy to the secondary side when it is high enough to power the load or to charge the high-capacity storage element.

There are several commercially-available integrated circuits (ICs) that can be used for the herein considered goal of optimally managing the power for medical wearable devices based on energy harvesting. In fact, most of the off-the-shelf solutions listed in Table 2 have multiple inputs for different energy harvesting sources, and can deliver currents of up to 100 mA (or up to about 500 mW for a 5 V output). Depending on the type of the used IC, the input voltage threshold (i.e., the minimum required output voltage from the harvesting device needed to “wake-up” the management electronics) can vary from the mV range to several tens of V, while the maximum input voltage is usually limited (clipped) by a protective shunt, and can be up to ~23 V. Some of the commercially-available solutions are produced together with embedded full-wave bridge rectifiers built from low-dissipation elements, so that low-power AC sources (such as the piezoelectric kinetic harvester devices) can be directly connected to their input pins. It is to be noted here also that, taking into account the working principle, as well as the properties of the used harvester and of the connected medical sensors, additional passive elements (resistors, capacitors and inductors) have to be added and optimized in order to efficiently use the harvested energy, and ensure undisturbed operation of the connected loads.

**Figure 16:**
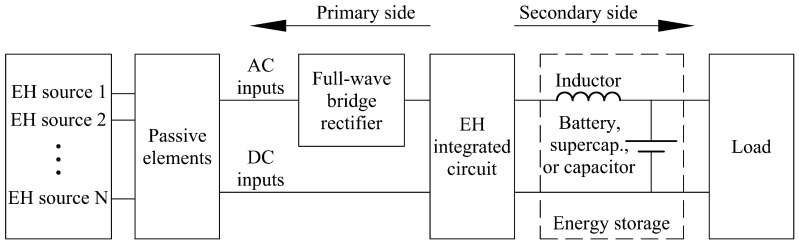
Generalized scheme of the energy harvesting power management electronics.

The generalized scheme of the power management electronics for medical wearable devices based on energy harvesting, with the corresponding main passive elements, is depicted in Figure 16. A detailed description of the optimization of such a circuitry, used in our research for a piezoelectric energy harvesting-based tire pressure monitoring system, is described in literature [10]. In order to achieve the maximal efficiency, the power requirements and sleep/measure/wake/transmission intervals of the factual tire pressure sensor with its corresponding data logging and data transmission circuitry, were experimentally determined in that case and used as input for the calculation of the passive elements of the energy management circuitry. In a different set-up, using a completely different energy harvesting principle, a similar approach to the optimization of the energy management electronics was also successfully demonstrated [11].

## 5. Conclusions and Outlook

The power requirements of sensors and associated data logging and transmission circuitry for wearable medical applications are thoroughly analyzed in this work. Based on this analysis, the numerical tools needed to assess the possibility to power such components by using piezoelectric kinetic energy harvesting devices are developed, and their main features, enabling the study of the respective complex dynamical electromechanical coupling behavior, are systematically analyzed, as well as validated by comparison to the experimental data. Since the considered class of energy harvesters is characterized by a narrow area of optimal operation around their eigenfrequencies, whereas the excitations generated by human motion are random, innovative design configurations are needed. Advancing on previous work reported in literature, novel designs of piezoelectric kinetic energy harvesters are thus conceived, modeled and scrutinized with the purpose of optimization and miniaturization, while broadening the usable bandwidths and maximizing the obtained powers, while also considering the respective strength constraints. A solution based on optimized segmented harvester’s geometries, on a frequency up-conversion excitation mechanism, and on appropriate power management electronics, suitable for wearable medical devices, is thus proposed.

**Figure 17:**
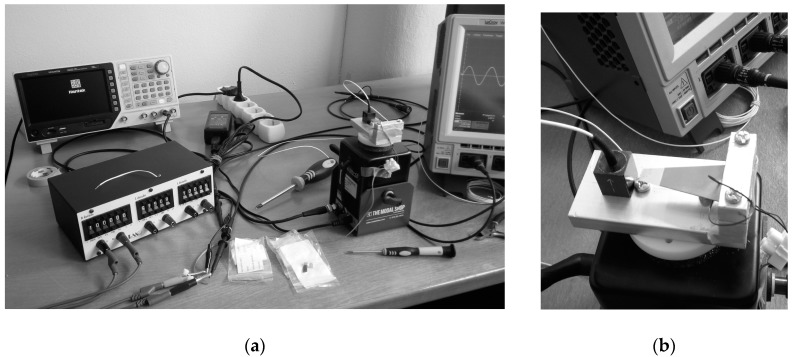
(**a**) Experimental set-up at the Brno University of Technology; (**b**) Detail of the trapezoidal piezoelectric kinetic harvester during the measurements.

**Figure 18:**
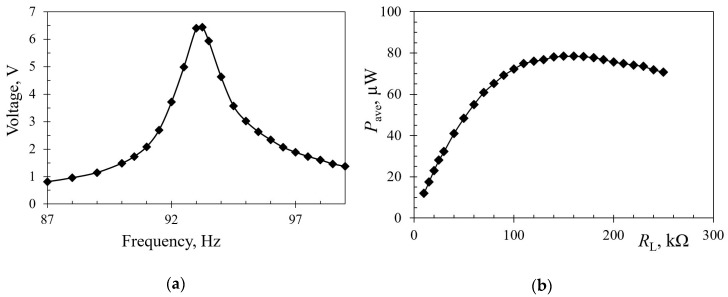
Preliminary experimental results for a trapezoidal cantilever: (**a**) Voltage and (**b**) Power spectra.

In order to validate the numerically-obtained results on the proposed design configurations, a thorough experimental analysis is currently being set up, and will be used next. It is based on a shaker, an accelerometer and a laser Doppler vibrometer. The set-up is being interfaced to a LabVIEW-based NI data acquisition system at the premises of the mentioned Precision Engineering Laboratory of the Faculty of Engineering of the University of Rijeka, Croatia [38]. Under development concurrently there is also a SPICE^®^ model of the complete system, i.e., including the harvester and the corresponding power management electronics, which should enable an easier optimization of the latter. In the meantime, preliminary measurements on a trapezoidal piezoelectric kinetic harvester with “dummy” loads and without a properly optimized power management electronics, have been carried out at the COST action CA18203 partnering institution, the Brno University of Technology, Czech Republic (Figure 17). The thus-obtained results are shown in Figure 18.

**Figure 19:**
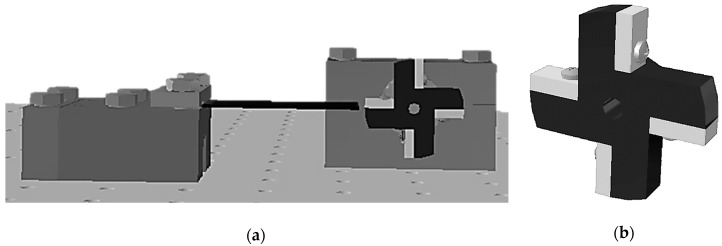
3D model of the frequency up-conversion experimental prototype: (**a**) Adjustable clamping mechanism with the rotational plucking device; (**b**) Detail of the excitation mechanism with exchangeable plectra.

Systematic experiments of controlled frequency up-conversion excitations of piezoelectric kinetic energy harvesters, conducted on an unloaded system, as well as on the system coupled to the proposed power management electronics with corresponding sensors (electrical loads) connected to its output, will thus be performed in Rijeka next. The design of the needed system, comprising an adjustable clamping mechanism, a plucking device apt to include exchangeable plectra of varying stiffness, and connected to an actuator with controllable rotation speed, is shown in Figure 19. The thus-attained results will be compared with the numerically-attained data, so as to allow a further development of innovative piezoelectric kinetic energy harvesting systems optimized for wearable applications in telemedicine and remote health monitoring. Finally, a practical application of the developed concepts in actual medical applications on patients, will allow developing original configurations of power sources for integrated autonomous wearable medical devices.

## Figures and Tables

**Table 1:** Power consumption of typical wearable devices and Internet of Things (IoT) components.

Device Device	Voltage	Power Consumption	Ref.
**Accelerometers**			
Analog, 300 mV/g, ADXL337	3.0 V	900 μW	[16]
Digital, 3.9 mg/LSB, ADXL345	2.5 V	350 μW	[16]
KX022 tri-axis (*—low power mode)	1.8–3.6 V	522 (36*) μW	[17]
**Temperature sensors**			
BD1020HFV −30 °C to +100 °C	2.4–5.5 V	38.5 μW	[17]
MAX30208 0 °C to +70 °C	1.7–3.6 V	241 μW	[18]
MCP9700 −40 °C to +150 °C	2.3–5.5 V	82 μW	[19]
**Heart rate monitors**			
Samsung Galaxy Gear Neo 2^®^ component	-	~50 mW	[20]
MAX30102 pulse oximetry/heart-rate monitor	1.8–3.3 V	˂1 mW	[18]
BH1790GLC optical heart rate sensor	1.7–3.6 V	720 μW	[17]
**Blood pressure sensors**			
Conformal ultrasonic device	-	~24 mW	[21]
CMOS Tactile Sensor	5 V	11.5 mW	[22]
3-Axis Fully-Integrated Capacitive Tactile Sensor	1.8–3.3 V	1.2–4.6 mW	[23]
**Blood glucose monitoring systems**			
IoT-based continuous glucose monitoring system	2.0 V	1 mW	[24]
Continuous glucose monitoring contact lens	~100 mV	˂1 μW	[25]
Implantable RFID continuous glucose monitoring sensor	1.0–1.2 V	50 μW	[26]
**Microphones**			
MEMS microphone, digital, ADMP441	1.8 V	2.52 mW	[16]
Electret condenser microphone, KEEG1542	2.0 V	1 mW	[16]
MEMS microphone, analog, ICS-40310	1.0 V	16 μW	[16]
**Pulse oximeter sensors**			
Reflective organic pulse oximetry sensing patch	3.3–5.0 V	68–125 μW	[27]
MAX30102 pulse oximetry/heart-rate monitor	1.8–3.3 V	˂1 mW	[18]
Ultra-low-power pulse oximeter with amplifier	5.0 V	4.8 mW	[28]
**A/D converters**			
AD7684 16-bit SAR 100 kS/s	2.7–5.0 V	15 μW	[16]
ADS1114 16-bit sigma-delta 0.860 kS/s	2.0–5.5 V	368 μW	[16]
DS1251 24-bit sigma-delta 20 kS/s	3.3–5.0 V	1.95 mW	[18]
**Signal processors**			
MC56F8006 Audio DSP, 16-bit 56800E	1.8–3.6 V	4282 μW/MHz	[16]
STM32L151C8 High-perf. MCU, 32-bit ARM Cortex-M3	1.7–3.6 V	540 μW/MHz	[16]
nRF52832 Bluetooth SoC, 32-bit ARM Cortex-M4	1.7–3.6 V	100 μW/MHz	[16]
**Wireless communication devices**			
RFID 13.56 MHz 860–960 MHz (range: 0–3 m)	5.0 V	200 mW	[29]
Bluetooth 2.4–2.5 GHz (range: 1–100 m)	-	2.5–100 mW	[29]
MICS 402–405 MHz (range: 0–2 m)	-	25 μW	[29]

**Table 2:** Typical off-the-shelf integrated circuits applicable to manage the power for medical wearable devices based on energy harvesting.

Device Type	Input Voltage	Output Voltage(s)	Inputs	Ref.
**Solar/piezoelectric kinetic/electro-magnetic energy harvesting devices**				
MB39C811	2.6–23 V DC/AC	1.5, 1.8, 2.5, 3.3, 3.6, 4.1, 4.5 and 5.0 V DC	2 AC, 1 DC	[11]
**Solar/piezoelectric kinetic/electro-magnetic energy harvesting devices**				
LTC3588-1	2.7–20 V DC/AC	1.8, 2.5, 3.3 and 3.6 V DC	2 AC, 1 DC	[10]
LTC3588-2	14–20 V DC/AC	3.45, 4.1, 4.5 and 5.0 V DC	2 AC, 1 DC	[51]
**Solar/thermo-electric/radio-frequency/piezoelectric kinetic energy harvesting devices**				
MAX17710	0.75–5.3 V DC	1.8, 2.3 and 3.3 V DC	2 DC	[18]
